# Exploring Factors Associated with Perceived Changes in Severity of Elder Abuse: A Population-Based Study of Older Adults in Korea

**DOI:** 10.3390/ijerph191610033

**Published:** 2022-08-14

**Authors:** Haesang Jeon, Jooyoung Kong

**Affiliations:** 1 Department of Social Welfare, Jeonju University, Jeonju-si 55069, Korea; 2 Sandra Rosenbaum School of Social Work, University of Wisconsin-Madison, 1350 University Avenue, Office 304, Madison, WI 53706, USA

**Keywords:** elder abuse, older Korean, perceived changes in severity of abuse

## Abstract

Elder abuse is a pressing problem that demands social attention in South Korea. This study aims to examine the characteristics of older adults and their family perpetrators that may influence the perceived severity of abuse by older adults using a nationally representative sample among older Koreans. We analyzed 952 community-dwelling older Koreans from a population-based survey of the Survey of Elderly Care and Welfare Need. The analytic sample of this study consisted of older adults who self-reported having been emotionally, physically, and financially abused or neglected by their family members or other primary caregivers. We used multinomial logistic regression models to predict perceived change in severity of abuse. Results showed that the abuse type and duration of abuse were significantly associated with the perceived change in the severity of abuse. Older victims’ age, being female, and being married were also associated with greater risk for increased severity of abuse relative to no change, while older adults’ better health status was associated with lower risk for increased severity of abuse. The findings of this study can help social work professionals identify older adults with heightened risk of abuse and protect the human rights of the most vulnerable aging population.

## 1. Introduction 

Elder abuse is a pressing problem that demands social attention and affects millions of individuals worldwide [1,2,3]. According to Roberto [4], elder abuse has a profound impact on the health and psychological well-being of older adults, resulting in increased morbidity and mortality rates. Mirroring this trend, elder abuse is also a pervasive phenomenon in South Korea (hereafter Korea). A recent nationwide elder abuse survey in 2017 showed that approximately 9.8% of older adults were victims of abuse or neglect [5]. This number is projected to increase owing to the rapid growth of the aging population in Korea [6]. The proportion of Koreans aged 65 years comprised 13.8% of the total population in 2017, and by 2025, it is projected to reach 20.8% [5]. Likewise, the number of formal elder abuse complaints filed with the Korea Elder Protection Agency also increased from 10,162 in 2013 to 13,309 in 2017, accounting for a 31% increase [7]. 

Greater involvement of families in caregiving due to inadequate formal services has been identified as one of the major risk factors for elder abuse [1]. Stress of caregiving has been reported to be a major cause of mistreatment in Korea [1]. According to Yan and Kwok [8], exposure to permanent stress and burden of care among caregivers may lead to potentially harmful and abusive behavior toward older adults. Likewise, about 89.9% of elder abuse took place in domestic settings in Korea, predominantly by the elder son followed by the spouse and daughter-in-law [5]. A study also showed that elder abuse victims had little or no choice but to continue to co-reside with their perpetrators despite the recurring abuse, leaving devastating after-effects on victims [9,10]. 

Although elder abuse has been increasingly recognized as a violation of human rights, it tends to remain hidden and unacknowledged among older Koreans [6,11]. According to Lee et al. [6], abuse is rarely disclosed by older Korean adults or is likely to be managed within the family due to cultural stigma. Recent studies have shown that Korean older adults who have low education, are currently single, Activities of Daily Living (ADL)-dependent, socially isolated, and co-reside with family perpetrators had an increased risk of abuse [11,12,13]. Contrarily, family perpetrators who live with older adults, are single, have mental disorders, have experienced abuse in the past by their parents, and are alcohol or substance abusers are more likely to engage in abuse [9,11,14].

### Perceived Severity of Elder Abuse and the Ecological Approach

To date, much of the research has focused on the prevalence of elder abuse, while little is known about older adults’ subjective perceptions of abuse. The majority of studies on elder abuse has used binary outcomes (occurrence of elder abuse vs. non-occurrence) and compressed the range and depth of abuse into a single category of positive abuse cases [15]. This type of approach is helpful in identifying the prevalence rates of elder abuse and risk factors that may lead to it; however, it oversimplifies the complex nature of abuse and provides little information about its dimensions, such as the severity of its perception. Moreover, the circumstances or the risk factors that determine the older adult victims’ appraisals of mistreatment as serious or benign are unknown [16]. Such information is important because, in the real world, elder abuse response interventions are not always able to remove older adults from potentially harmful environments [10]. In most circumstances, elder abuse victims have the right to choose the least restrictive intervention to preserve family relationships and to avoid exposing family perpetrators to the justice system [17]. This is in line with older Korean adults, as they choose family relations and refuse to participate in abuse interventions [10]. Thus, a more responsive measure of elder abuse, such as the severity of abuse, would help identify abuse intervention models that would alleviate victims’ abusive circumstances [17].

The ecological approach is an optimal theory for explaining the perceived severity of elder abuse. The theory identifies factors embedded within different dimensions of surroundings that may influence the severity of elder abuse [15]. It treats aging as the outcome of the reciprocal interaction between a person and the significant contexts of life, such as family and peer relationships [15]. Given the complexity of elder abuse and the limited empirical research on its perceived severity, an applied ecological approach could best address the requirements toward a better contextual understanding of elder abuse. According to Burnes et al. [16], the severity of elder abuse is the product of individual vulnerabilities that contribute to status inequality and power imbalances within the relationships caused by age-associated impairments in older adults. Moreover, the characteristics of perpetrators and the history of violence between older adults and perpetrators in the past can also contribute to the intensification of their abuse severity. Although limited in numbers, a recent population-based elder abuse study uncovered that emotional abuse was appraised less seriously among older victims who were functionally impaired, heavily dependent, or co-residing with the family perpetrator [16]. Higher neglect severity is associated with functional impairment, younger age, low income, low education, and living only with the family perpetrator [15]. 

The severity of elder abuse is an important dimension that requires attention. According to Burnes et al. [15], elder abuse is most accurately understood along a continuum of severity. Moreover, the severity framework is a more suitable tool in practice, as community-based adult protective services aim to reduce further harm in accordance with the victim’s preferred resolution, which often includes the risk of ongoing revictimization [17]. Such information is germane to understand why some older adults remain in potentially harmful situations while others seek or accept help from the outside [16]. To address this gap in the literature, the current study aimed to examine the characteristics of older adults and their family perpetrators that may influence the perceived severity of abuse among older adults using a nationally representative sample of older Koreans. Guided by Burnes and colleagues’ [16] interpretation of the ecological approach in elder abuse severity, this study identified elder abuse as a function of the interacting characteristics of older adults and perpetrators and victim–perpetrator relationships. 

## 2. Research Methods

### 2.1. Data Sources

We used data from the 2009 Survey of Elderly Care and Welfare Need (SECW) conducted by the Korea Institute of Health and Social Welfare. The SECW is a nationally representative dataset that has been widely recognized for providing robust statistics about elder abuse among older Korean adults. The sampling process first began by dividing the nation into general districts where the majority of housing included detached houses and apartment districts. Next, the districts were divided into 6 stratums to represent 7 metropolitan cities and suburbs. Based on the 2008 National Consensus, 300 districts were again selected and were stratified by age, gender, and geographic location. In total, 8607 community-dwelling adults aged 65 were contacted, and 6745 older adults completed the survey, which yielded a survey response rate of 78%. In carrying out the survey, trained interviewers visited their assigned respondents to conduct face-to-face individual interviews from November 2009 to April 2010. 

The analytic sample of this study consisted of 952 older adults who self-reported having been emotionally abused, physically abused, financially abused, or neglected by their family members or other primary caregivers. About one-fifth of respondents reported having been abused by a spouse, 56% abused by other family members, such as their biological children or their spouse, and 1% abused by a non-family member, such as friends or neighbors. The remaining 21% were neglected and did not specify perpetrators. Among 975 older adults who reported the experience of psychological, physical, and financial abuse and neglect, we excluded 23 cases who experienced the abuse “once” and did not report changes in the perceived severity of abuse. The survey was approved by Statistics Korea and followed the protocols to protect the research participants.

### 2.2. Measures

#### 2.2.1. Elder Abuse

In total, 28 questions were asked to identify older adults who experienced elder abuse by families and/or other caregivers in dimensions of emotional, physical, and financial abuse and neglect. If respondents reported a ‘*yes*’ to any of the 28 items, follow-up questions were asked about the timing of the abuse (e.g., occurred in the past 12 months), severity, duration, and perpetrator characteristics. If respondents reported a ‘*yes*’ to more than one dimension of victimization, respondents reported follow-up information about the abuse that lasted the longest. 

*Types of Abuse.* Four different abuse types were assessed, including emotional abuse, physical abuse, financial abuse, and neglect. First, emotional abuse was measured by seven items that asked respondents whether their family members or other primary caregivers had ever (a) ignored them or avoided making conversations with them; (b) yelled, screamed, or cursed at them; (c) expressed verbally that they no longer wanted to live with them, (d) isolated them from meeting friends or attending activities; (e) threatened to kill them; (f) did not respond to what they requested or forced them to do things that they refused to do; and (g) terrorized or menaced them. Physical abuse was measured by eleven items that asked respondents whether their family members or other primary caregivers had ever (a) pinched or bitten them, or pulled their hair; (b) pushed or shoved them, (c) beat them up; (d) punched or hit them with something that could hurt; (e) choked them; (f) forced them to stay in the house or kept them out of the house; (g) bound their hands and feet to prevent them from moving around; (h) forced them to take unnecessary medicine or injections; (i) forced them to do work that they did not want to do or they were not able to do; (j) forced them to display sexual body parts and they felt shame; and (k) sexually harassed them. Financial abuse was measured by four items that asked respondents whether (a) their family members or other primary caregivers had ever used or took their money or property without their permission, (b) their family members or other primary caregivers did not comply with an agreement so that the respondents experienced financial loss, (c) their financial documents (i.e., bank statements, bequeathing statements) were falsified or forcibly signed, and (d) their family members or other primary caregivers controlled the management of their money or property. Lastly, neglect was measured by six items that asked respondents whether family members or other primary caregivers had ever (a) not provided necessary help with activities of daily living, such as eating or household chores; (b) not taking any actions to equip the house; (c) not provided any financial help to support basic necessities; (d) not provided necessary medical care; (e) abandoned them; and (f) been out of reach. Based on the 28 items, a variable was created with four categories: (1) emotional abuse, (2) physical abuse, (3) financial abuse, (4) neglect.

*Perceived Change in Severity of Abuse.* The key dependent variable of this study was the change in severity of abuse, which was measured by an item that asked respondents the extent to which the severity of abuse had changed since the first time it had started. This item had five response choices: (1) much weakened, (2) somewhat weakened, (3) did not change, (4) somewhat strengthened, (5) much strengthened. For the multinomial analysis, this variable was recoded to have three categories: (1) somewhat or much weakened, (2) did not change, (3) somewhat and much strengthened. 

*Duration of Abuse.* The duration of abuse was assessed by an item that asked respondents how long the abuse lasted. The item had seven response choices: (1) less than a month, (2) 1 month–less than 6 months, (3) 6 months–less than a year, (4) 1 year–less than 3 years, (5) 3 years–less than 5 years, (6) 5 years–less than 10 years, (7) 10 years and more. 

#### 2.2.2. Characteristics of Older Adults

*Socio-demographic characteristics.* We considered socio-demographic characteristics of respondents, including their gender (*male*, *female*), marital status, age (*years*), individual income, and self-reported health status. Marital status was originally indicated by six categories, namely, (a) having a partner or spouse, (b) widowed, (c) divorced, (d) separated, (e) never married, (f) others, which was then dichotomously recoded into (0) non-married and (1) married. The unit of individual income was 10,000 won (approximately 10 U.S. dollars). To deal with skewness, this variable was top-coded with 500 (5,000,000 won; 5000 US dollars) being the highest value, and 0 as the lowest value. Self-reported health status was assessed by a four-point Likert scale ranging from (1) very poor/poor to (4) very good.

*Social isolation.* Respondents’ levels of social isolation were assessed by two aspects: frequency of contact with friends/neighbors, and frequency of participating in social activities. First, frequency of contact with friends/neighbors was measured by an item that asked respondents how often, in the past 12 months, they contacted their friends or neighbors who they can share their problems or concerns with. The item had five response choices: (1) never, (2) less than once in 3 months, (3) once or twice a month, (4) once a week, (5) more than once a week. Second, frequency of participating in social activities was measured by an item that asked respondents how often, in the past 12 months, they participated in social gatherings or organizations (e.g., informal groups, religious groups). The item had five response choices: (1) never, (2) less than once in 3 months, (3) once or twice a month, (4) once a week, and (5) more than once a week.

#### 2.2.3. Characteristics of Perpetrators

*Socio-demographic characteristics.* Respondents reported several characteristics of perpetrators, including their gender (*male*, *female*), marital status, age, educational attainment, and financial status. Perpetrators’ marital status was originally indicated by six categories, namely, (a) having a partner or spouse, (b) widowed, (c) divorced, (d) separated, (e) never married, (f) others, which was then dichotomously recoded into (0) non-married and (1) married. Perpetrators’ ages were asked, namely, whether they were in their (1) 20s–30s, (2) 40s, (3) 50s, or (4) 60s or older. Perpetrators’ educational attainment was indicated by eight categories ranging from being illiterate to post-college education, which was recoded into four categories: (1) no formal education—completed elementary school, (2) completed middle school, (3) completed high school, and (4) completed college and above. Financial status was indicated by (1) very poor, (2) poor, (3) fair, and (4) well-off/very well-off. 

*Past and current adversities.* Respondents reported perpetrators’ other characteristics, including their current problem behaviors, burden of caregiving, and a history of being raised in the violent family environment. First, a question was asked whether perpetrators exhibited problem behaviors related to alcohol/drug abuse or cognitive impairment, such as dementia, which was assessed by a binary response (1 = yes, 0 = no). Respondents also reported their perception about the extent to which the perpetrator felt the burden of providing care to the respondents. The item had five response choices: (1) not at all, (2) to a small extent, (3) to a moderate extent, (4) to a great extent, and (5) to a very great extent. Lastly, a question was asked whether the perpetrator was raised in a violent family environment while growing up, which was assessed by a binary response (1 = yes, 0 = no). 

### 2.3. Analysis Methods

Using Stata 15 (i.e., using the mlogit command), we estimated a multinomial logistic regression model predicting perceived change in severity of abuse. The dependent variable had three categories: (1) somewhat or much weakened, (2) did not change, and (3) somewhat or much strengthened, and the *did not change* category served as the base outcome. When estimating multinomial logistic regression models using the mlogit command in Stata, relative risk ratios (RRRs) are produced to indicate the risk of the outcome occurring in the given comparison group relative to the risk of the outcome occurring in the reference group. The complete cases without missing values were provided by 83.7% of the data. The variable with the most missing values was perpetrator’s history of violent family environment (n = 69, 7.25%). Missing values were imputed using a multivariate imputation by chained equations (MICE) procedure using the ICE command [18,19].

## 3. Results

### 3.1. Descriptive Statistics

Table 1 presents descriptive statistics of key variables. About one quarter of respondents (n = 234) reported that the severity of abuse had increased, and 16% reported that the severity had declined (n = 154). Three quarters of respondents experienced emotional abuse (n = 712), and 21% reported that they had experienced neglect (n = 199). The average duration of abuse was 3 years–less than 5 years (*M* = 5.49, *SD* = 1.43). About 70% of respondents were female (n = 653) and about half were married or had a partner (n = 450). On average, respondents were 72.71 years old (*SD* = 5.88) and reported a *fair* health status based on the four-point Likert scale (*M* = 2.56, *SD* = 0.93). On average, respondents participated in social activities about *once or twice a month* (*M* = 2.94, *SD* = 1.67). In terms of perpetrators’ characteristics, on average, perpetrators were in their 40s (*M* = 2.53, *SD* = 1.06). The majority of perpetrators was male (56%) and married (72%). About 7% of perpetrators exhibited problem behaviors, such as substance abuse (n = 65) and reported a history of having been raised in a violent family environment (n = 63). 

### 3.2. Factors Associated with Changes in Intensity of Abuse 

Table 2 presents the summary of multinomial logistic regression analysis predicting perceived change in severity of abuse. First, older adults who experienced neglect, compared to those who experienced emotional abuse, were less likely to report that the severity of abuse had decreased (RRR = 0.08; 95% CI [0.03, 0.24], *p* < 0.001). Older adults who experienced a longer duration of abuse were more likely to report declined severity of abuse (RRR = 1.20; 95% CI [1.03, 1.39], *p* < 0.05). Older adults who perceived perpetrators’ burden of caregiving were less likely to report declined severity (RRR = 0.78; 95% CI [0.66, 0.91], *p* < 0.01). 

Additionally, older adults who experienced neglect, compared to those who experienced emotional abuse, were less likely to report that the severity of abuse had increased (RRR = 0.25; 95% CI [0.15, 0.42], *p* < 0.001). Respondents with older age were more likely to report increased severity of abuse (RRR = 1.04; 95% CI [1.01, 1.07], *p* < 0.01). In addition, older men, compared to older women, were less likely to report increased severity (RRR = 0.57; 95% CI [0.38, 0.85], *p* < 0.01). Married older adults, compared to non-married older adults, were more likely to report increased severity (RRR = 1.67; 95% CI [1.12, 2.48], *p* < 0.05). Respondents with better health status were less likely to report increased severity (RRR = 0.83; 95% CI [0.69, 0.99], *p* < 0.05). Older adults who perceived perpetrators’ current problem behaviors were more likely to report increased severity (RRR = 2.59; 95% CI [1.35, 4.96], *p* < 0.01).

## 4. Discussion

One of the notable contributions of the current study is the identification of the significant factors at different levels that were associated with older victims’ perceived change in the severity of abuse in Korea. The ecological framework guided us to investigate the characteristics of abuse in older adults, and their perpetrators, which helped enhance a holistic understanding of the risks associated with elder abuse victimization. 

In relation to the characteristics associated with abuse, we found that the severity of neglect was less likely to change in cases of emotional abuse. This result is similar to a previous study of older Koreans, where neglect by family members was repetitive and continued until the person from the outer circle, perhaps a social worker, intervened [20]. Considering the finding that a longer duration of abuse was associated with a greater likelihood of reporting decreased severity, the issue of elder abuse may be more chronic and maintained at a steady level within families, which could lead to detrimental cumulative impacts on older adults [21]. This finding may also reflect a normalized long-standing pattern of abuse by the family perpetrator. 

In relation to characteristics associated with older victims, this study showed that older victims are more likely to report a greater intensity of elder abuse as compared to the younger victims. This result is contrary to previous findings from the United States, which consistently showed that younger older adults are at a greater risk of elder abuse since they often co-reside with a spouse or with adult children who are most likely to be abusers [20,22]. A possible explanation is that old age may result in greater dependence on others for care, possibly for extended periods, which may place older adults at a higher risk of abuse. Considering the significant role of family members in caring for older adults in Korea, being old or experiencing a prolonged period of having been cared for may result in a greater severity of elder abuse by family members. 

Older victims’ gender predicted perceived change in the severity of elder abuse in such a way that male victims were less likely to report that abuse had become stronger compared to females. One convincing explanation for this finding is that the result may reflect the patriarchal culture in Korea, where men hold primary power in society and within family relationships [23]. With this in mind, older women who are at the lower end of the hierarchy in the family may encounter abuse more frequently than their male counterparts. Thus, intervention strategies should adopt gender consciousness and focus on strengthening the resilience of female victims to prevent the recurrence of abuse. 

The finding that being married is associated with a greater risk of increased severity is also incongruent with the findings of Western studies. For instance, a study of older adults in the United States found that marriage decreased the risk of emotional and physical abuse [22]. Burnes et al. [24] explain that marriage lasting into late adulthood could be protective by virtue of having survived previous life challenges together. However, contrary to these previous findings, our findings are more in line with previous studies with older Korean adults—that those who are currently married are more likely to report a higher intensity of abuse [9]. A possible explanation for this is that abuse by a spouse could have been present throughout the marriage and continued later in life. Further studies on elder abuse should consider intimate partner violence in older adults. 

In relation to the characteristics associated with perpetrators, their caregiving burden was associated with a greater severity of abuse toward older adults. This result supplements previous findings that delineated the relationship between caregiver burden and abuse [4,21,23]. Prior studies have shown that caregiving can become stressful, and the burden on caregivers can lead to potentially harmful or abusive behaviors toward older recipients [4]. Lee and colleagues [6] stated that caregivers who are exposed to permanent stress and burden of care are at a high risk of abusive behavior toward older adults if they have the sole responsibility for their aging relatives. In this regard, the present study emphasizes the importance of practice and policy interventions that can alleviate the caregiving burden of family caregivers. 

To reduce caregiving burden and elder abuse, the Korean government introduced a universal long-term care insurance program in 2007 to provide financial support and assistance with daily living activities (e.g., household chores) to older persons with extensive functional impairment. However, such services are limited to older adults who live alone or with severe disabilities, and family caregivers are excluded from these services. Contrarily, numerous studies have consistently highlighted the positive effects of family caregiver interventions to prevent elder abuse [3,8,14]. For example, counseling services and support groups have successfully alleviated the caregiving burden in many cases [3]. Thus, expanding caregiver support programs will be effective in alleviating the caregiving burden, which could help prevent elder abuse.

As expected, perpetrators’ behavioral problems were associated with greater severity of elder abuse. Drug and alcohol abuse, along with perpetrators’ gambling, have been well documented as contributing factors to elder abuse [9,11,25]. Considering the significant association between perpetrators’ behavioral problems and a greater intensity of abuse, interventions should target family perpetrators along with older adults as service recipients from the beginning. Collaborative efforts between local community resources, services, and agencies to protect older adults may help mitigate perpetrators’ behavioral problems toward older family members. Moreover, increasing participation and retention of family perpetrators in the program is necessary to increase the success of the intervention program [14].

## 5. Conclusions

Our findings highlighted that the type and duration of abuse were significantly associated with perceived changes in abuse severity. In addition, older victims’ age, being female, and being married were associated with a greater risk for increased severity of abuse relative to no change, while better health status was associated with a lower risk of increased severity of abuse. We also found that perpetrators’ problematic behaviors were associated with a greater risk of increased severity, and their burden of caregiving was associated with a lower risk of decreased severity.

However, the current study has several limitations. First, this study was based on self-reports of older adults regarding abuse and perpetrators, which were subjected to recall and social desirability biases. It is possible that older adults may have underreported abuse because of the embedded stigma associated with abuse among family members [10]. Second, potentially important risk factors and confounders, including formal assistance in caregiving and housekeeping, were unavailable for analysis. Assistance in caregiving or household chores could minimize caregiver burden, which in turn lowers abuse [21]. Another plausible explanation is that the presence of an outsider could help caregivers become aware of their abusive behavior and be less likely to engage in those behaviors [21]. Lastly, the current study was unable to include perpetrators‘ occupations, which may determine their lifestyle habits leading to elder abuse.

Despite these limitations, this study contributes to the understanding of older Koreans’ experiences of abuse within their families. To the best of our knowledge, this is the first attempt to examine the changes in the severity of elder abuse in the context of relationships between older adults and perpetrators using a nationally representative sample of older Koreans. The findings of this study can assist social work professionals to identify older adults with a heightened risk of abuse and to protect the human rights of the most vulnerable aging population. We recommend concerted efforts for policy change and practice interventions that can widen support resources and networks for older adults and promote positive interactions and functioning between family members and older adults.

## Figures and Tables

**Table 1:** Summary statistics of study sample and key variables (N = 952).

	N	%	Mean (SD)	Min/Max
Perceived change in severity of abuse				
Declined	154	16.18	-	-
No change	563	59.14	-	-
Increased	234	24.58	-	-
Types of abuse				
Emotional abuse	712	74.79	-	-
Physical abuse	18	1.89	-	-
Financial abuse	23	2.42	-	-
Neglect	199	20.90	-	-
Duration of abuse ^a^	-	-	5.49 (1.43)	1/7
**Characteristics of Victims of Elder Abuse**				
Age	-	-	72.71 (5.88)	65/94
Gender				
Male	299	31.41	-	-
Female	653	68.59	-	-
Marital status				
Married	450	47.27	-	-
Non-married	502	52.73	-	-
Self-rated health ^b^	-	-	2.56 (0.93)	1/4
Income (10,000 Won)	-	-	134.07 (117.54)	0/500
Frequency of contact with friends/neighbors ^c^	-	-	2.52 (1.78)	1/5
Frequency of participating in social activities ^c^	-	-	2.94 (1.67)	1/5
**Characteristics of Perpetrators**				
Age ^d^	-	-	2.53 (1.06)	1/4
Gender				
Male	537	56.41	-	-
Female	415	43.59	-	-
Marital status				
Married	685	71.95	-	-
Non-married	252	26.47	-	-
Educational attainment ^e^	-	-	2.55 (1.07)	1/4
Financial status ^f^	-	-	2.39 (0.92)	1/4
Problematic behaviors (e.g., substance use)	65	6.83		
Burden of caregiving ^g^	-	-	3.47 (1.40)	1/5
History of violent family environment	63	6.62		

*Notes*. Descriptive statistics presented as mean and standard deviation for the continuous variables, and frequency and percentage for the categorical variables. ^a^ Duration of abuse was coded as (1) less than a month, (2) 1 month–less than 6 months, (3) 6 months–less than a year, (4) 1 year–less than 3 years, (5) 3 years–less than 5 years, (6) 5 years–less than 10 years, (7) 10 years and more. ^b^ Self-reported health was coded as (1) very poor/poor, (2) fair, (3) good, (4) very good. ^c^ Frequency of contact with friends/neighbors and participating in social activities were coded as (1) never, (2) less than once in 3 months, (3) once or twice a month, (4) once a week, (5) more than once a week. ^d^ Perpetrator’s age was coded as (1) 20’s-30’s, (2) 40’s, (3) 50’s, (4) 60’s older. ^e^ Educational attainment was coded as (1) elementary level, (2) graduated middle school, (3) graduated high school, (4) some-college—graduate level. Financial status was coded as (1) very poor, (2) poor, (3) okay, (4) well-off/very well-off. ^f^ Educational attainment was coded as (1) elementary level, (2) graduated middle school, (3) graduated high school, (4) some-college–graduate level. ^g^ Burden of care was coded as (1) strongly disagree, (2) disagree, (3) neither disagree nor agree, (4) agree, and (5) strongly agree.

**Table 2:** Factors associated with perceived change in severity of abuse (N = 952).

	Severity Declined ^a^	Severity Increased ^a^
	RRR [95% CI]	
Types of abuse		
Emotional abuse (reference)	-	-
Physical abuse	1.12 [0.96, 1.03]	0.81 [0.24, 2.71]
Financial abuse	1.99 [0.68, 5.85]	0.54 [0.16, 1.77]
Neglect	0.08 [0.03, 0.24] ***	0.25 [0.15, 0.42] ***
Duration of abuse	1.20 [1.03, 1.39] *	1.01 [0.90, 1.14]
Characteristics of Older Adults		
Age	1.00 [0.97, 1.04]	1.04 [1.01, 1.07] **
Male	0.70 [0.43, 1.14]	0.57 [0.38, 0.85] **
Married	1.64 [0.99, 2.74]	1.67 [1.12, 2.48] *
Self-rated health	0.88 [0.71, 1.09]	0.83 [0.69, 0.99] *
Income	1.00 [1.00, 1.00]	1.00 [1.00, 1.00]
Frequency of contact with friends/neighbors	1.06 [0.95, 1.19]	1.00 [0.91, 1.10]
Frequency of participating in social activities	0.91 [0.81, 1.04]	0.92 [0.83, 1.02]
Characteristics of Perpetrators		
Age	1.06 [0.83, 1.35]	1.03 [0.84, 1.27]
Male	1.09 [0.69, 1.72]	1.05 [0.72, 1.52]
Married	1.45 [0.84, 2.51]	0.98 [0.65, 1.48]
Educational attainment	0.86 [0.69, 1.07]	0.94 [0.78, 1.13]
Financial status	0.85 [0.66, 1.08]	0.85 [0.69, 1.06]
Problematic behaviors (e.g., substance use)	1.25 [0.55, 2.87]	2.59 [1.35, 4.96] **
Burden of caregiving	0.75 [0.64, 0.88] **	1.02 [0.89, 1.17]
History of violent family environment	0.88 [0.40, 1.94]	1.32 [0.69, 2.49]
Constant	0.45 [0.02, 9.73]	0.06 [0.01, 0.73] *

*Notes*. ^a^ Reference category: severity did not change. Significance level was denoted as * *p* < 0.05, ** *p* < 0.01, *** *p* < 0.001. RRR = relative risk ratio; CI = confidence interval.

## Data Availability

The data presented in this study are available at the Korea Institute for Health and Social Affairs, https://data.kihasa.re.kr/kihasa/kor/databank/DatabankDetail.html (accessed on 1 June 2020).
